# A Synthetic Lethal Screen Identifies DNA Repair Pathways that Sensitize Cancer Cells to Combined ATR Inhibition and Cisplatin Treatments

**DOI:** 10.1371/journal.pone.0125482

**Published:** 2015-05-12

**Authors:** Kareem N. Mohni, Petria S. Thompson, Jessica W. Luzwick, Gloria G. Glick, Christopher S. Pendleton, Brian D. Lehmann, Jennifer A. Pietenpol, David Cortez

**Affiliations:** Department of Biochemistry, Vanderbilt University School of Medicine, Nashville, Tennessee, United States of America; University of South Alabama Mitchell Cancer Institute, UNITED STATES

## Abstract

The DNA damage response kinase ATR may be a useful cancer therapeutic target. ATR inhibition synergizes with loss of ERCC1, ATM, XRCC1 and DNA damaging chemotherapy agents. Clinical trials have begun using ATR inhibitors in combination with cisplatin. Here we report the first synthetic lethality screen with a combination treatment of an ATR inhibitor (ATRi) and cisplatin. Combination treatment with ATRi/cisplatin is synthetically lethal with loss of the TLS polymerase ζ and 53BP1. Other DNA repair pathways including homologous recombination and mismatch repair do not exhibit synthetic lethal interactions with ATRi/cisplatin, even though loss of some of these repair pathways sensitizes cells to cisplatin as a single-agent. We also report that ATRi strongly synergizes with PARP inhibition, even in homologous recombination-proficient backgrounds. Lastly, ATR inhibitors were able to resensitize cisplatin-resistant cell lines to cisplatin. These data provide a comprehensive analysis of DNA repair pathways that exhibit synthetic lethality with ATR inhibitors when combined with cisplatin chemotherapy, and will help guide patient selection strategies as ATR inhibitors progress into the cancer clinic.

## Introduction

DNA damaging chemotherapy agents such as cisplatin are standard of care treatments for many solid tumors including triple-negative breast cancer (TNBC) and non-small cell lung cancer (NSCLC). These agents work by placing an increased dependency on DNA damage responses for survival and proliferation. Mutations in DNA repair genes are frequent in TNBC and NSCLC, and genomic studies indicate significant genome instability in a subset of TNBC suggesting defects in DNA repair [1–5]. TNBC often has a good initial response to chemotherapy including platinum drugs but patients almost invariably relapse and can develop resistance [6, 7]. NSCLC patients receive platinum as a first-line drug and typically survive less than one year [8].

The DNA damage response kinase ATR (ATM- and Rad3-related) coordinates many of the cellular responses to DNA damage ATR is necessary to stabilize stalled replication forks and allow fork restart after damage [9]. In the absence of ATR, stalled replication forks collapse into double strand breaks, which can lead to genomic rearrangements or cell death [10, 11]. ATR activation is also required to slow the cell cycle to allow time for repair, through phosphorylation of its effector kinase CHK1 [9]. ATR is an essential kinase, and many cancer cells have an increased dependence on ATR to compensate for oncogene-induced replication stress [12–14].

Selective ATR inhibitors have been described by Vertex Pharmaceuticals [15, 16] and AstraZeneca [17] and are currently in phase I clinical trials in combination with DNA damaging chemotherapy drugs or radiation therapy. To identify in which genomic context ATR inhibitors might best be used as a monotherapy we previously conducted a synthetic lethal siRNA screen to identify genes that when inactivated sensitized cells to ATR inhibition. Inactivation of the ERCC1-XPF endonuclease as well as loss of known ATR pathway proteins and DNA replication proteins strongly sensitized cells to ATR inhibition [18]. ATR inhibition is also synthetically lethal with loss of XRCC1 and ATM as well as overexpression of Cyclin E [18–21].

ATR inhibition synergizes with DNA damaging chemotherapy drugs such as cisplatin and gemcitabine to kill cancer cells [15, 19]. ATR inhibition has shown efficacy in a mouse model of pancreatic cancer in combination with gemcitabine, and in patient-derived lung tumor xenografts in combination with cisplatin [16, 22]. Thus, clinical trials will include combination treatments with an ATR inhibitor and cisplatin, gemcitabine, or etoposide (ClinicalTrials.gov: NCT02157792). Here we report the first systematic siRNA synthetic lethality screen combining ATR inhibition and cisplatin treatment to look for more targeted applications of the ATR inhibitor when combined with chemotherapy. As expected, we identified the ATR pathway, DNA replication genes, and ERCC1-XPF. There was no added benefit of combining ATRi and cisplatin in either homologous recombination (HR)-deficient or mismatch repair (MMR)-deficient cells. We did find that loss of translesion DNA polymerases and 53BP1 hyper-sensitizes cells to ATRi/cisplatin combination treatment. Since inactivating mutations are found in these genes in cancers, our data suggests therapeutic value for combined ATRi/cisplatin in these settings.

## Materials and Methods

### Cells and reagents

U2OS and HCT-116 were obtained from Stephen Elledge, August, 2002. MDA-MB-468 (HTB-132), HCC1806 (CRL-2335), BT549 (HTB-122), H157 (CRL-5802), and A549 (CCL-185) were obtained from the ATCC and maintained as previously described [18]. The following cell lines were previously described: BRCA2 defective and complemented VC8 cells [23], HCT-116 + chromosome 3 [24], hec59, and hec59 + chromosome 2 [25]. MDA-MB-468 cisplatin-resistant cells were generated by continual selection in cisplatin, and maintained in media supplemented with 3μM cisplatin. The cisplatin-resistant cells were grown without cisplatin for 7 days prior to the start of each experiment to control for any effects of cisplatin treatment. The ATR inhibitor (ATRi) VE-821 [19] was synthesized by the Vanderbilt Institute for Chemical Biology Chemical Synthesis Facility. The PARP inhibitor (PARPi) BMN673 [26] was purchased from Selleck Chemicals. Cisplatin was purchased from Calbiochem.

### Synthetic lethal siRNA screen

The synthetic lethal siRNA screen was done as previously described [18]. Briefly, we utilized a custom siRNA library targeting 240 known DNA repair and replication genes with 4 siRNAs per gene in a 96-well format. U2OS cells were transfected with the siRNA library and split into four 96-well plates 72 hours after transfection. Cells were then either left untreated or treated with 1μM ATRi, 0.5μM cisplatin, or 1μM ATRi and 0.5μM cisplatin. Cell viability was determined after an additional 72 hours using alamar blue (Invitrogen). The percent viability of each sample was determined by comparing the drug treated sample to the untreated sample for each gene to control for any siRNA-specific effects on cell growth rates. Robust z-scores were calculated using the median and the median absolute deviation of the log_10_(percent viability) values. The values presented are the mean robust z-scores from three independent transfections. Genes selected as exhibiting synthetic lethal relationships with any of the drug treatments have at least 2 siRNAs with robust z-scores less than -1.3. The ATRi only arm of the screen was previously published [18].

### RNA interference and cell viability assays

All siRNA transfections were performed as previously described with 10nM siRNA and Dharmafect 1 (Invitrogen) [18]. The following siRNA sequences were used: siREV3L-2 GAAGUUAUCUGGCUGCUUU, siREV3L-4 CAAAGAUGCUGCUACAUUA, si53BP1-2 GGACAAGUCUCUCAGCUAU, si53BP1-3 GAUAUCAGCUUAGACAAUU. The non-targeting siRNA was Qiagen All Star Negative control. The short-term cell viability assays were done as previously described [18]. Briefly, cells were plated in 96 well plates 72 hours after transfection with siRNA and then treated with drugs for 72–96 hours as indicated in the figure legends. Cell viability was compared to an untreated control after subtracting blank well values from all of the data. The maximum DMSO concentration at the highest dose of drug was 0.01% and had no effect on cell growth. Clonogenic assays with U2OS cells were performed as previously described [27]. In all cell viability assays the values represent the mean (n = 3) and error bars represent the standard deviation of one experiment. All experiments were performed at least twice.

### Synergy Analysis

Bliss independence log synergy volumes (μM^2^%) were calculated with using MacSynergy II and reported at the 95% confidence interval for one replicate (n = 3) [28]. Peaks on the graph correspond to synergy and the higher the peak the stronger the degree of synergy. Isobologram analysis was performed as described [29, 30]. Fractional inhibitory concentrations were determined by dividing the IC_50_ of ATRi with a fixed concentration of cisplatin or PARPi by the IC_50_ of ATRi alone (x-coordinate). The y-coordinate is the fixed concentration of cisplatin/PARPi divided by the IC_50_ of cisplatin/PARPi alone. The solid diagonal line on the graph represents additivity and values beneath the line represent synergy. IC_50_ values were determined using Prism version 6.

### Immunofluorescence analysis

Performed and quantified as previously described [27].

## Results

### ATR inhibition synergizes with cisplatin and resensitizes cisplatin-resistant cancer cells

**Fig 1:**
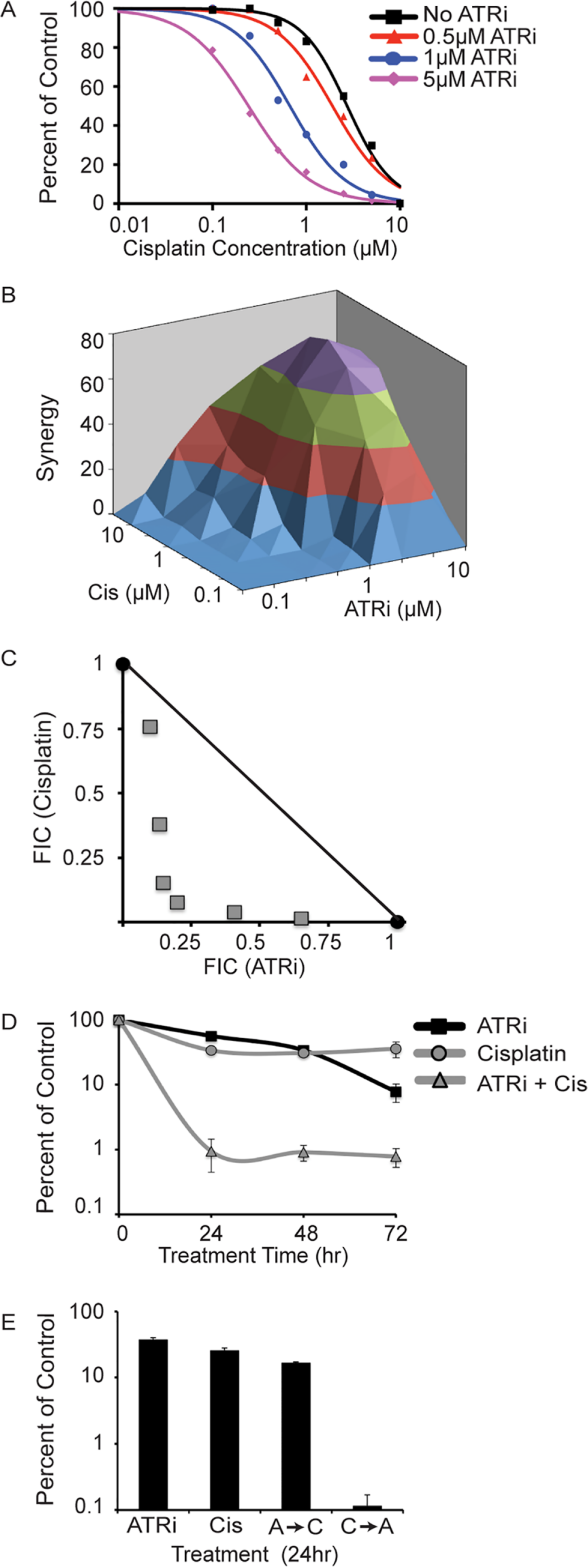
ATR inhibition sensitizes cells to cisplatin. (A and B) U2OS cells were treated with increasing doses of cisplatin or the ATR inhibitor (ATRi) alone or in combination for 72 hours. Cell viability was determined with alamar blue and reported as a percentage of the untreated control cells. Analysis of synergy between cisplatin and ATRi using Bliss Independence (B) and isobologram analysis (C) as described in the materials and methods. (D) U2OS cells were treated with 1μM cisplatin, 1μM ATRi, or both (ATRi + Cis); cells were released into media without drugs after 24, 48, or 72 hours and allowed to form colonies. (E) Cells were treated with 1μM cisplatin or 1μM ATRi for 24hr, ATRi (24hr) followed by cisplatin (24hr) A→C, or cisplatin (24hr) followed by ATRi (24hr) C→A. Error bars in all panels are standard deviation (n = 3).

ATR inhibitors (ATRi) exhibit synergy with cisplatin in killing HCT-116 colon cancer cells suggesting combinations of ATRi and cisplatin may be useful therapeutically [15, 19]. Consistent with this result, increasing amounts of ATRi reduce the concentration of cisplatin required to kill U2OS osteosarcoma cells and both agents exhibit marked synergy in short-term viability assays (Fig 1A and 1B). Synergy is reported in a 3-dimensional graph with the two single-agents (ATRi or cisplatin) plotted in the X and Z dimensions and a Bliss independence log synergy volume plotted in the Y dimension. Peaks on the graph correspond to the degree of synergy with higher peaks indicating larger amounts of synergy [28]. Synergy was also evaluated using isobologram analysis (Fig 1C). The fractional inhibitory concentrations (FIC) of ATRi and cisplatin are shown on the x-axis and y-axis respectively. Values beneath the solid line represent synergy. To confirm these observations in long-term viability assays, U2OS cells were treated with ATRi, cisplatin, or ATRi/cisplatin combination for 24, 48, or 72 hours and allowed to form colonies for 14 days (Fig 1D). At the concentrations used, both ATRi and cisplatin treatment alone slightly reduced the ability of cells to form colonies. The ATRi/cisplatin combination treatment reduced colony formation by almost two orders of magnitude more than the single treatments alone. Thus, there is large synergy between ATRi and cisplatin in long-term viability assays. In addition, almost all of the cell killing effect of the combined treatment is imparted during the first 24 hours of exposure, as longer incubation times do not further reduce the colony forming ability of the cells.

Next we asked whether the order of treatment is important for the synergy. U2OS cells were either treated with ATRi or cisplatin alone for 24 hours, or treated with ATRi for 24 hours followed by cisplatin for 24 hours or vice versa (Fig 1E). Cells treated with ATRi, cisplatin, or ATRi followed by cisplatin exhibit a 2-5-fold reduction in the ability to form colonies. Strikingly, cells treated with cisplatin followed by ATRi exhibited a 1000-fold reduction in their ability to form colonies. Together these data indicate that maximum synergy is observed when cells are treated with ATRi and cisplatin simultaneously, or when cisplatin treatment precedes ATRi. No synergy was observed when ATRi treatment preceded cisplatin.

**Fig 2:**
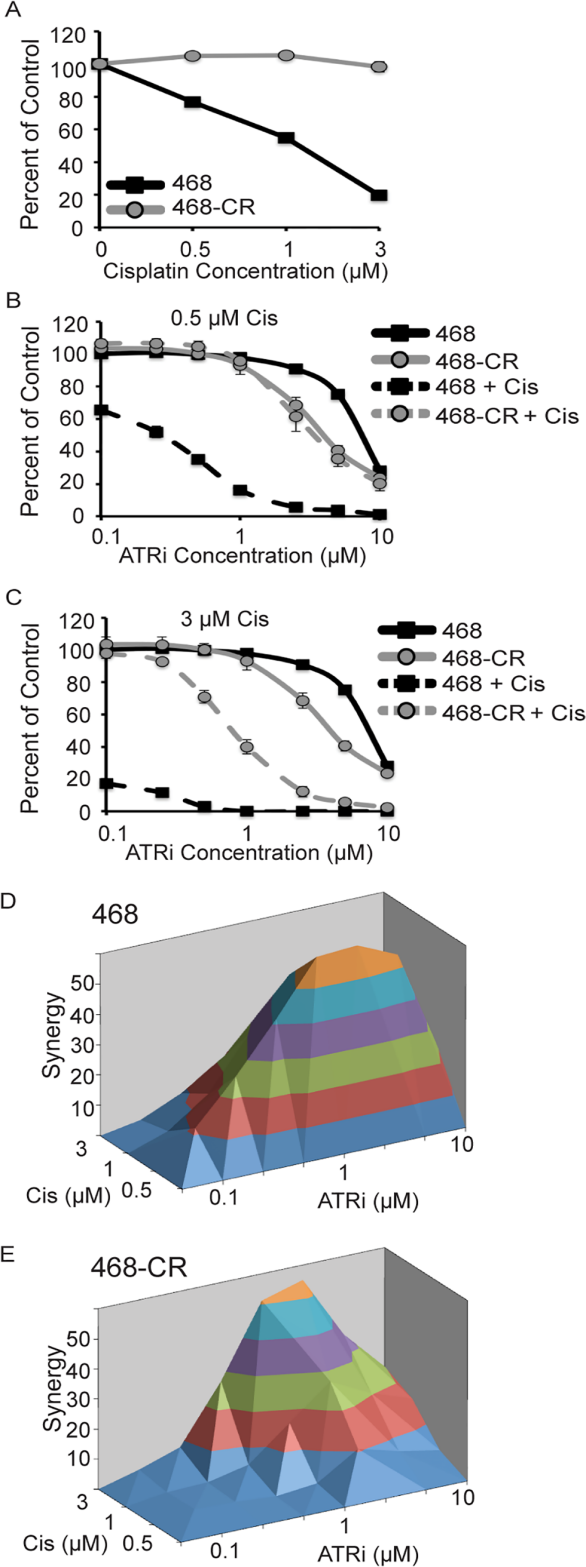
ATR inhibition resensitizes cisplatin-resistant cancer cells to cisplatin. (A) MDA-MB-468 (468) and MDA-MB-468 cisplatin-resistant (468-CR) cells were treated with increasing doses of cisplatin for 96h prior to measuring cell viability with alamar blue. (B and C) MDA-MB-468 and MDA-MB-468 cisplatin-resistant cells were treated with ATRi alone or ATRi and cisplatin at 0.5μM (B) or 3μM (C) for 96h prior to measuring cell viability with alamar blue. (D and E) Bliss independence synergy between cisplatin and ATRi in MDA-MD-468 (D) and MDA-MB-468 cisplatin-resistant cells (E). Error bars in all panels are standard deviation (n = 3).

The strong synergy observed between ATRi and cisplatin led us to hypothesize that ATRi might resensitize cisplatin-resistant cancers. To test this idea, we generated cisplatin-resistant MDA-MB-468 triple negative breast cancer (TNBC) cells by continuous stepwise exposure to increasing concentrations of cisplatin (Fig 2A). These cells were treated with increasing concentrations of ATRi alone, or in combination with 0.5μM or 3μM cisplatin (Fig 2B and 2C). As expected, the parental cell line exhibited synergy with 0.5μM cisplatin and was completely killed by 3μM cisplatin (Fig 2D). The cisplatin-resistant cell line exhibited no synergy with 0.5μM cisplatin but did exhibit synergy with 3μM cisplatin, albeit not to the level of the parental cells (Fig 2E). Thus, ATR inhibition was able to resensitize cisplatin-resistant cells to cisplatin, but these cells were still not as sensitive as the parental cells. In these experiments, synergy was observed when the dose of cisplatin begins to have an effect on cell viability (5–10 percent reduction in viability) and maximal synergy was observed when the dose of cisplatin reduced cell viability by 25–50 percent of the untreated control. Unexpectedly, the cisplatin-resistant cell line was slightly more sensitive to the ATR inhibitor alone suggesting that the mechanism of resistance to cisplatin made the cells more dependent on ATR signaling for survival.

### Identification of synthetic lethal interactions with ATRi/cisplatin combination treatment

**Fig 3:**
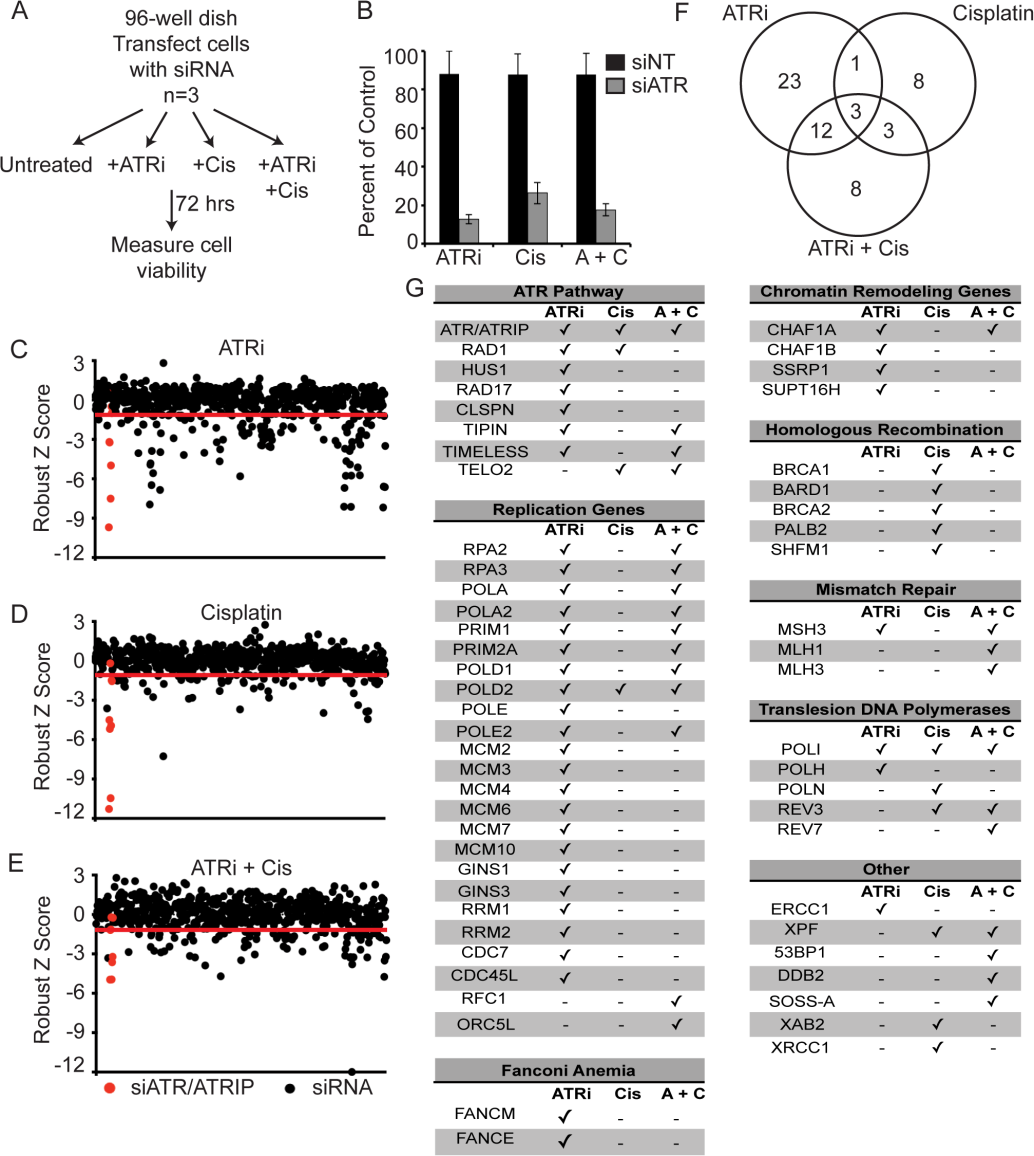
siRNA screen identifies synthetic lethal interactions with cisplatin and ATR inhibitor treatment. (A) Schematic of the siRNA screen. U2OS cells were transfected with siRNAs and then either left untreated or treated with 1μM ATRi, 0.5μM cisplatin, or ATRi and cisplatin. Cell viability was measured with alamar blue. (B) Viability of the non-targeting (NT) and ATR siRNA controls using the screen conditions. The values represent the mean ± SD of the three independent replicates of the screen. (C-E) The robust z-scores of treated compared with untreated cell viability were determined for each siRNA in the library for ATRi (C), cisplatin (D), and ATRi and cisplatin (E). The red circles represent the 8 unique siRNAs targeting ATR and ATRIP present in the library and the red line indicates a robust z score of -1.3. (F) Summary of the overlap of synthetic lethal relationships with ATRi, cisplatin, or ATRi and cisplatin. (G) Complete list of genes that exhibit a synthetic lethal relationship with ATRi, cisplatin, or ATRi and cisplatin. Genes with 2 or more siRNAs with robust z-scores of less than -1.3 are considered as synthetic lethal. The ATRi single-agent screen was previously published and included for comparison to the other drug treatments [18].

To identify genetic determinants of the observed synergy between ATRi and cisplatin, we conducted three synthetic lethal siRNA screens to search for genes, that when depleted, sensitized cells to ATRi or cisplatin alone as well as ATRi/cisplatin combination treatment. We used a custom siRNA library that contains 240 known DNA repair and replication genes with 4 individual siRNAs per gene in separate wells of 96-well plates [18]. U2OS cells were transfected with the siRNA library. 72 hours later each 96-well plate was split into four 96-well plates and either left untreated, or treated with 1μM ATRi, 0.5μM cisplatin, or a combination of 1μM ATRi and 0.5μM cisplatin for 72 hours (Fig 3A). Cell viability was measured with alamar blue, and the percent viability of each siRNA was calculated by comparing the alamar blue value of the treated siRNA compared to the untreated siRNA. This method controls for any siRNA-specific effects on cell growth. The drug doses were chosen such that they had a minimal effect on the cell viability of the non-targeting siRNA and had a maximal effect on the ATR siRNA controls added to each plate of the screen (Fig 3B). We have proposed that lower doses of ATRi are sufficient to kill ATR-depleted cells because less ATRi is needed to completely inhibit the remaining ATR protein [18]. All three drug treatments were analyzed independently, and the percent viability was used to calculate a robust z-score for each drug treatment as described in the materials and methods. The screen was completed in triplicate, and the mean robust z-scores were plotted for each siRNA for each of the drug treatments (Fig 3C–3E). The internal positive control siRNAs targeting ATR and ATRIP are highlighted in red and the solid red line indicates the threshold cutoff used to select siRNAs. S1 Dataset presents the complete results of all three screens. Genes with at least 2 siRNAs having a robust z-score of -1.3 or less were selected as exhibiting synthetic lethal relationships. A relatively modest robust z-score cutoff was selected since this is a biased library of DNA damage response and replication proteins that we expect to have a higher frequency of synthetic lethal interactions than a random library.

The three screens identified genes that exhibited synthetic lethal relationships in one or more of the drug combinations and in several distinct DNA repair pathways (Fig 3F and 3G). The ATRi arm of the screen was previously published and included here for comparison to the combination treatment screen [18]. As previously reported, the largest family of genes identified that exhibit synthetic lethality with ATRi alone were ATR pathway genes and DNA replication genes, including a previously unreported synthetic lethal interaction with ribonucleotide reductase (*RRM1* and *RRM2*). We also identified chromatin remodelers, Fanconi Anemia pathway genes, translesion DNA polymerases, and *ERCC1* [18]. *ATM* and *XRCC1* did not reach our cutoff with more than one siRNA and were not included in the list of synthetic lethal interactions. These interactions were previously identified using null and complemented cell lines as well as small molecule inhibitors [19, 20]. It is possible that complete silencing/inhibition is required to observe the synthetic lethality reported and was not accomplished with the siRNA used in our library.

Using cisplatin as a single agent, we observed synthetic lethality with HR genes as expected since this is a major mechanism required to repair cisplatin adducts. Cells deficient in *ATR*, translesion DNA polymerase pathways, XPF (*ERCC4*), *XAB2*, and *XRCC1* are also hypersensitive to cisplatin.

The combined ATRi/cisplatin sensitivity screen was carried out at the same doses of ATRi and cisplatin that were used in the single drug screens. This screen also identified ATR pathway genes and a subset of DNA replication genes, which are likely present based on their synthetic lethality with ATRi alone. Novel gene families found in the combined treatment include mismatch repair genes, additional translesion DNA polymerases, XPF (*ERCC4*), *TP53BP1*, *DDB2*, and SOSS-A (*INTS3*). With the combination of ATRi/cisplatin treatment, *ATM*, *RAD50*, and *RAD54*L approach our significance cutoff threshold with one siRNA for each yielding a z-score less than -1.3 and a second siRNA with a robust z-score less than -0.9.

### Synthetic relationships between HR or MMR and ATRi/cisplatin

**Fig 4:**
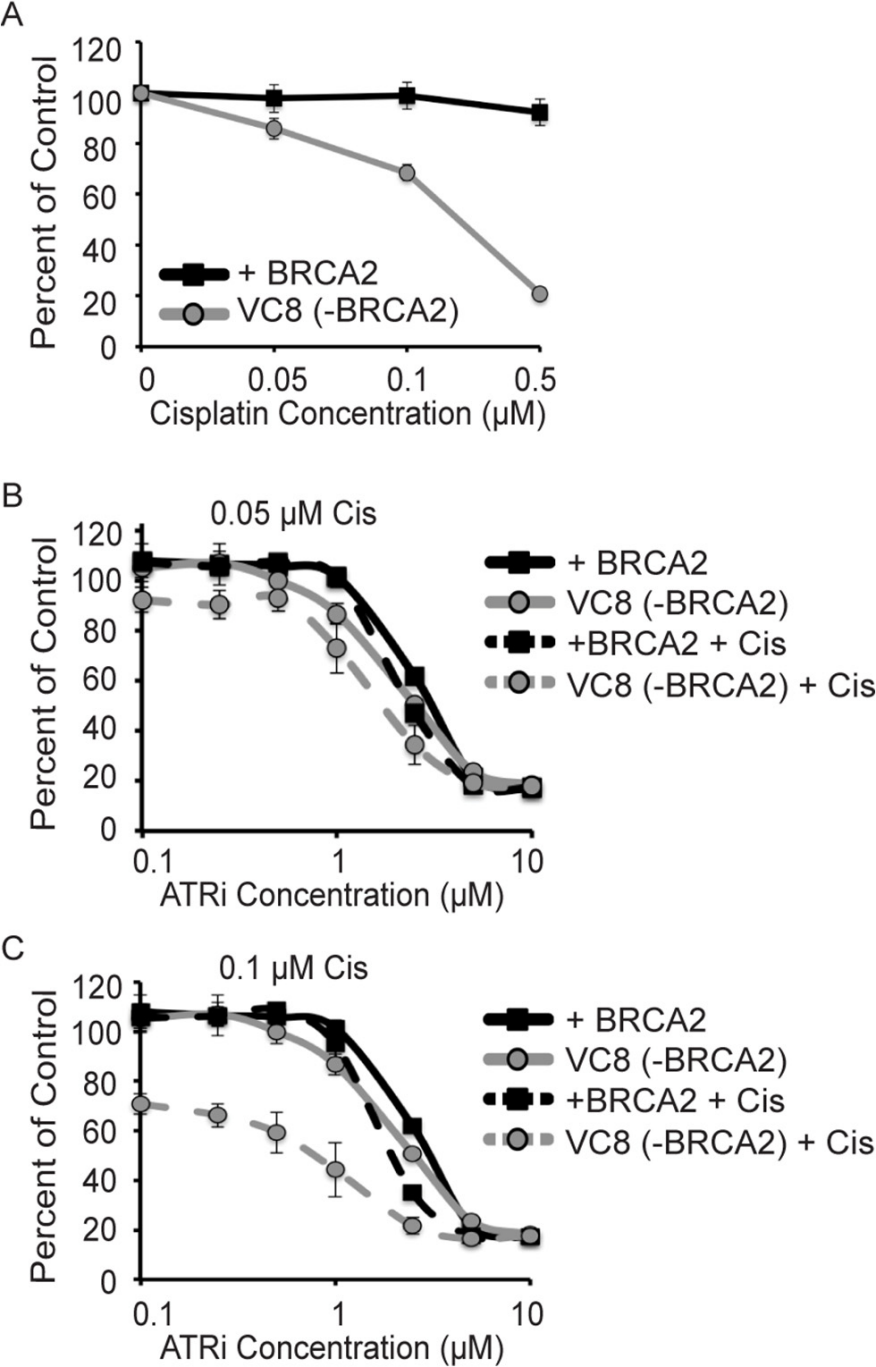
Loss of homologous recombination is not synthetic lethal with ATRi/cisplatin. BRCA2-deficient VC8 cells or VC8 cells complemented with a BRCA2 expression vector were treated with ATRi, cisplatin, and ATRi/cisplatin combination. (A) Sensitivity of BRCA2-deficient cells to cisplatin. (B and C) Sensitivity of BRCA2-deficient cells to ATRi alone and ATRi with either 0.05 or 0.1μM cisplatin. Cell viability was measured with alamar blue after 72 hours and reported as a percent of the untreated control cells. Error bars are standard deviation (n = 3).

Interestingly, loss of HR genes did not cause synergy with ATRi/cisplatin even though it is synergistic with cisplatin alone. We verified this result using BRCA2-deficient VC8 cells. These cells are extremely sensitive to cisplatin compared to isogenic cells engineered to re-express wild-type BRCA2 (Fig 4A). However, they are not hypersensitive to ATRi alone and the combination therapy of ATRi/cisplatin did not result in any synergistic killing at low or moderate cisplatin doses (Fig 4B and 4C). The large amount of death seen in the high dose cisplatin combination treatment is nearly all due to the enhanced toxicity of cisplatin in HR-defective cells. The lack of synthetic lethality may be because inhibition of ATR reduces the efficiency of HR so no further synergy is observed in HR-deficient backgrounds [31].

**Fig 5:**
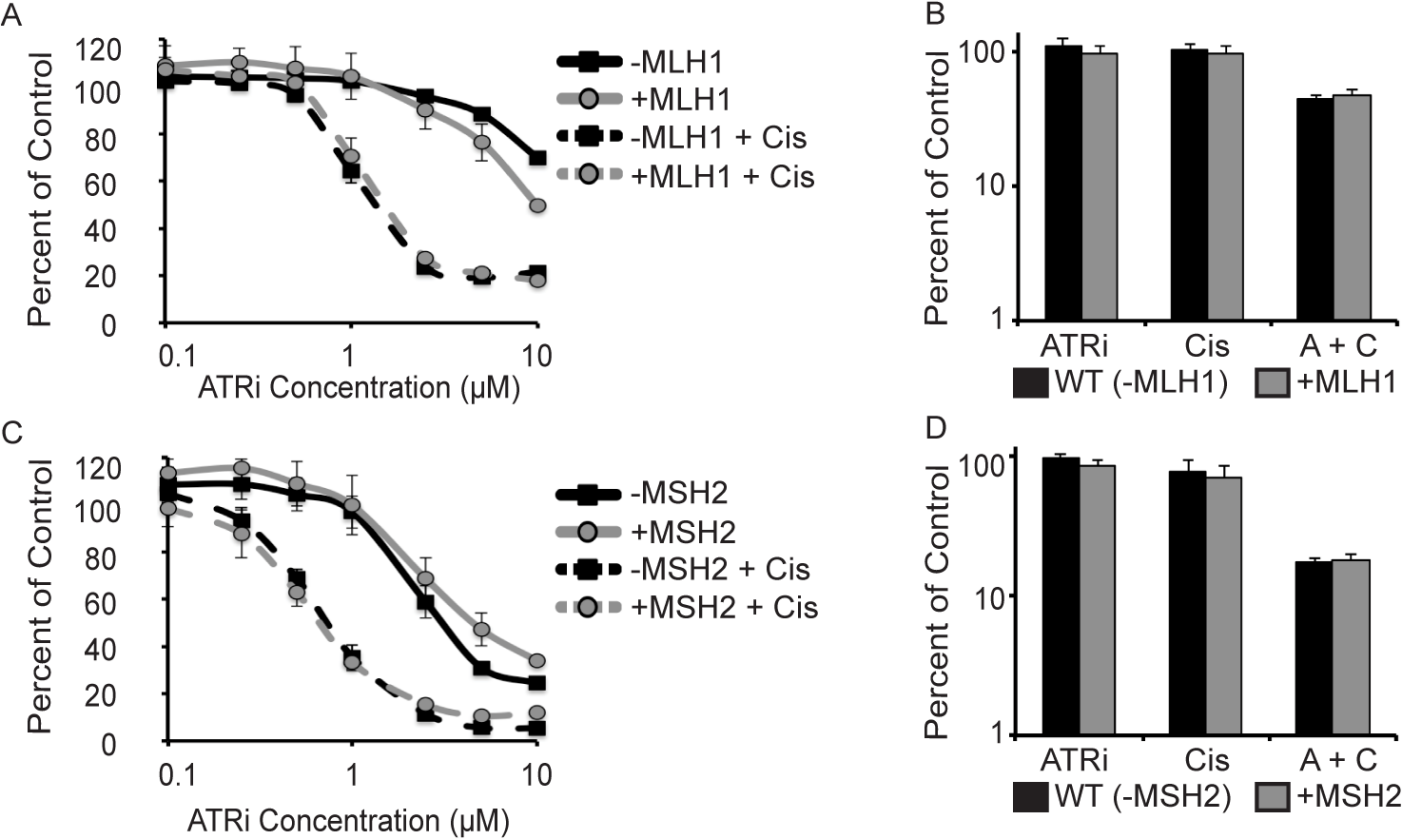
Loss of mismatch repair is not synthetic lethal with ATRi/cisplatin. (A and C) Cells were treated with increasing doses of ATRi alone or in combination with 0.5μM cisplatin for 72 hours. Cell viability was measured with alamar blue and reported as a percent of the untreated control cells for each cell line. (B and D) Cells were treated with 1μM ATRi, 0.5μM cisplatin, or both (A + C); cells were released into media without drugs after 24 hours and allowed to form colonies. (A and B) MLH1-deficient HCT-116 cells and HCT-116 cells complemented with MLH1. (C and D) MSH2-deficient HEC59 cells and HEC59 cells complemented with MSH2. Error bars in all panels are standard deviation (n = 3).

Several MMR genes exhibited synthetic lethality with the ATRi/cisplatin combination treatment in the screen. Since MMR-deficiency is a common feature of many cancers, we sought to verify that tumor cells with MMR-deficiency are hypersensitive to combined ATRi/cisplatin treatments. The colon cancer cell line HCT-116 is MMR-deficient due to the lack of MLH1. Surprisingly, when compared to HCT-116 + Chromosome 3 in which MLH1 expression has been restored [24], we found no difference in sensitivity to ATRi alone or the combination of ATRi/cisplatin in both short-term and long-term viability assays (Fig 5A and 5B). We also tested the role of MMR in HEC59 endometrial cancer cells, which are deficient in MSH2 and consequently do not express MSH3 or MSH6. These cells did not exhibit any synthetic lethality with ATRi alone or the combination of ATRi/cisplatin compared to the complemented cell line in both short-term and long-term viability assays (Fig 5C and 5D). Together these data indicate that mismatch repair is not a useful indicator of synthetic lethality with ATRi or ATRi/cisplatin combination treatment. Thus, several DNA repair pathways exhibit no synthetic lethality with ATR inhibition or the combination of ATR/cisplatin including HR and MMR.

### ATR inhibition is synthetic lethal with PARP inhibition

**Fig 6:**
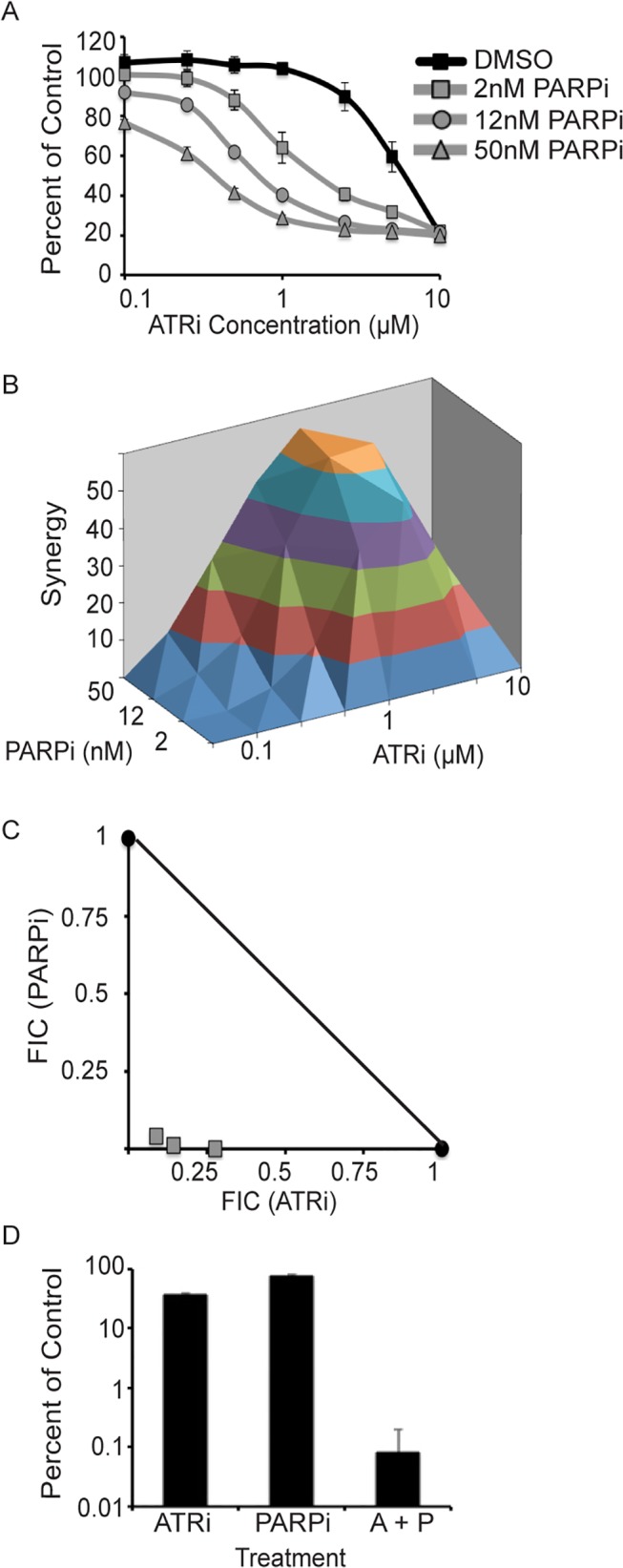
ATR inhibition is synthetic lethal with pharmacologic inhibition of PARP. (A) U2OS cells were treated with increasing doses of ATR and PARP inhibitors for 96 hours. Cell viability was measured with alamar blue and reported as a percent of the untreated control. Synergy between ATR and PARP inhibition using Bliss Independence (B) and isobologram analysis (C) as described in the materials and methods. (D) Cells were treated with 1μM ATRi, 2nM PARPi, or both (A + P) and cells were released into media without drugs after 72 hours and allowed to form colonies. Error bars in all panels are standard deviation (n = 3).

PARP inhibitors exhibit synthetic lethality with loss of HR, and are currently in clinical trials for treatment of BRCA-deficient tumors [32–34]. *PARP1*, *PARP2*, and *PARP4* were in our library and did not cause synthetic lethality with any of the drug treatments indicating that genetic loss of PARP does not sensitize cells to ATR inhibition. However, ATR was identified as the third highest scoring gene in a screen looking for synthetic lethal interactions with PARP inhibition [35] and ATR inhibition sensitized ovarian cancer cells to the PARP inhibitor Veliparib [36]. PARP inhibition can yield different results as compared to genetic loss of PARP genes due to the multiple PARPs in cells and the need for trapping PARP complexes on DNA for cell killing [34, 37–39]. U2OS cells treated with increasing doses of BMN673 significantly reduced the amount of ATRi needed to kill cells and exhibited marked synergy over a wide range of doses in short-term viability assays (Fig 6A–6C). This observation was even more pronounced in long-term colony forming assays where combination treatment of ATRi and BMN673 reduced the ability of cells to form colonies by over 1000-fold (Fig 6D). Similar results were obtained in HCT-116 cells.

### Loss of REV3 and 53BP1 sensitizes cancer cells to ATRi and cisplatin

Additional genes identified in the screen as exhibiting strong synthetic lethality with ATRi/cisplatin combination were *REV3L* and *TP53BP1*. *REV3L* was the fourth highest scoring gene (after *POLD2*, *ATR*, and *ATRIP*) identified. REV3 is the catalytic subunit of the translesion (TLS) polymerase ζ. The structural subunit of Pol ζ, REV7, was also identified as synthetic lethal with the ATRi/cisplatin combination with 2 out of 4 siRNAs. TLS is divided into two steps, insertion of a nucleotide opposite a damaged base and extension after the insertion [40]. Extension after the insertion by several TLS polymerases is often carried out by Pol ζ. This polymerase-switching event requires REV1. 3 of 4 *REV1* siRNAs caused synthetic lethality with ATRi/cisplatin (robust z-scores of -1.9, -1.0, and -0.9), although the magnitude of two of them just missed our significance threshold. The appearance of both subunits of Pol ζ and the polymerase required for loading it strongly suggests loss of this complex creates hypersensitivity to ATRi/cisplatin.

**Fig 7:**
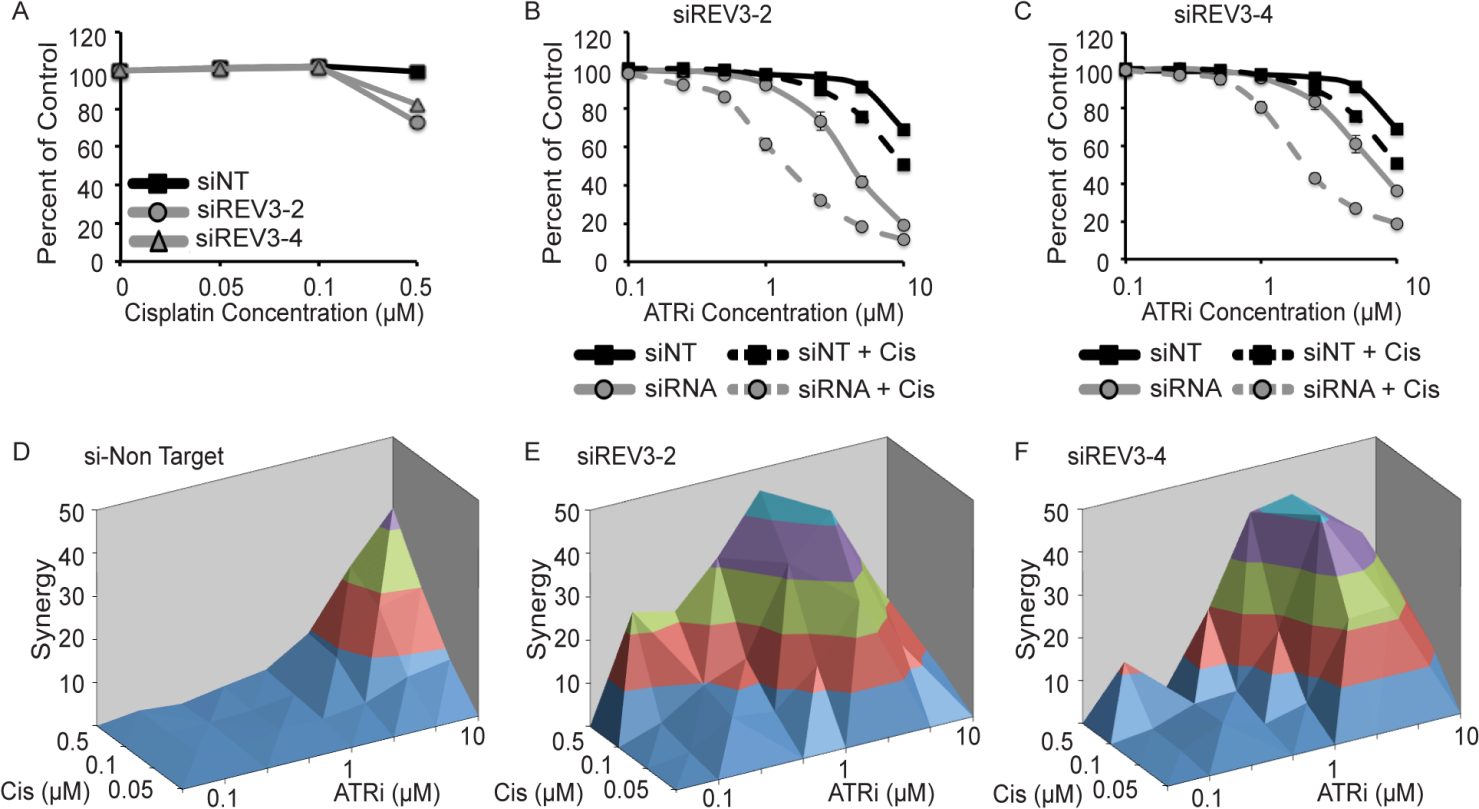
Loss of REV3 is synthetic lethal with ATRi and cisplatin. (A-F) H157 NSCLC cells were transfected with non-targeting siRNA (siNT) or two siRNAs targeting REV3 (number 2 and 4 refer to specific sequences described in the materials and methods). Cells were then treated with ATRi, cisplatin, and ATRi and cisplatin. Cell viability was determined with alamar blue and reported as a percent of the untreated control cells. (A) Sensitivity of REV3 knockdown cells to cisplatin. (B and C) Sensitivity of REV3 knockdown cells to ATRi and ATRi with 0.1μM cisplatin. Bliss independence synergy between ATRi and cisplatin in control (D) and REV3 knockdown cells (E and F). Error bars in all panels are standard deviation (n = 3).

To validate the results obtained in the screen and assess their generalizability we knocked down REV3 using two specific siRNAs in the NSCLC cell line H157. H157 cells depleted of REV3 were slightly more sensitive to high concentrations of cisplatin than cells expressing the non-targeting siRNA (Fig 7A). REV3-depleted cells were then treated with increasing doses of ATRi alone, or in combination with cisplatin. Depletion of REV3 moderately sensitized H157 cells to ATRi alone with both siRNAs (Fig 7B and 7C). Increased synthetic lethality was observed when cells were treated with ATRi/cisplatin combination (Fig 7B and 7C). In contrast, H157 cells transfected with the non-targeting siRNA exhibited synergy with ATRi and cisplatin only at high doses of ATRi/cisplatin combination (Fig 7D). Cells depleted of REV3 exhibited large amounts of synergy over a wide dose range of ATRi and cisplatin (Fig 7E and 7F). Maximal synergy was observed at the dose of cisplatin just before it exhibits any single-agent killing. We also observed similar results by colony forming assay (S1 Fig) and short-term viability assays with other NSCLC and TNBC cancer cell lines (S2–S4 Figs).

After REV3, *TP53BP1* was the second highest scoring gene that was not an ATR pathway or a DNA replication gene. 53BP1 is a DNA damage response protein with roles in double-strand break repair. 53BP1 prevents resection at double-strand breaks and influences the pathway choice for DNA repair by promoting non-homologous end joining [41–43]. 53BP1 is also required to protect chromosomal fragile sites and other under-replicated regions during mitosis for eventual repair in the subsequent G1 [44, 45].

**Fig 8:**
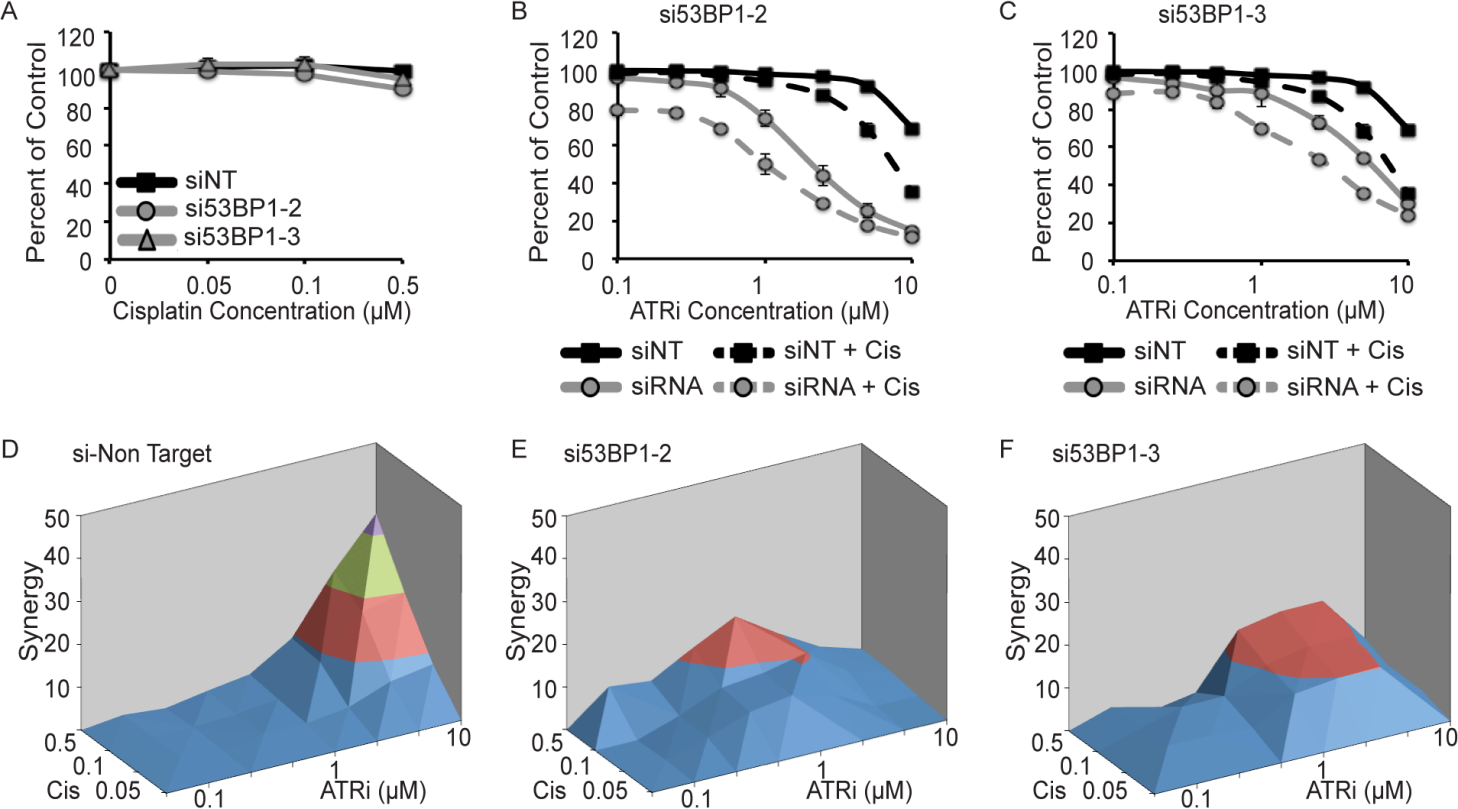
Loss of 53BP1 is synthetic lethal with ATRi and cisplatin. (A-F) H157 NSCLC cells were transfected with non-targeting siRNA (siNT) or two siRNAs targeting 53BP1 (number 2 and 3 refer to specific sequences described in the materials and methods). Cells were then treated with ATRi, cisplatin, and ATRi and cisplatin. Cell viability was determined with alamar blue and reported as a percent of the untreated control cells. (A) Sensitivity of 53BP1 knockdown cells to cisplatin. (B and C) Sensitivity of 53BP1 knockdown cells to ATRi and ATRi with 0.5μM cisplatin. Bliss independence synergy between ATRi and cisplatin in control (D) and 53BP1 knockdown cells (E and F). Error bars in all panels are standard deviation (n = 3).

To generalize the 53BP1 genetic relationship with ATRi/cisplatin combination, we analyzed reduction of 53BP1 in the H157 NSCLC cell line. Depletion of 53BP1 had a minor effect on the sensitivity of cells to single-agent cisplatin treatment (Fig 8A). 53BP1-depleted cells did exhibit marked sensitivity to ATRi treatment alone with both siRNAs tested (Fig 8B and 8C). Cell viability was further reduced with the combined treatment of ATRi/cisplatin. As observed earlier, combination treatment with ATRi/cisplatin exhibited synergy only at high doses of both drugs in cells transfected with the non-targeting siRNA (Fig 8D). Although there was a large decrease in cell viability, only modest effects on synergy were caused by 53BP1 silencing (Fig 8E, 8F and S5 Fig). This is most likely due to the large effect of ATR inhibition and 53BP1 depletion alone in this cell line. No synergy was observed in the other NSCLC cancer cell line tested, A549 (S6 Fig). However marked synergy was observed in the TNBC cell line HCC1806 with both siRNAs tested and in BT549 cells with one siRNA (S7 and S8 Figs). Thus, there are likely other genetic modifiers influencing the genetic relationships between 53BP1 and ATRi/cisplatin.

**Fig 9:**
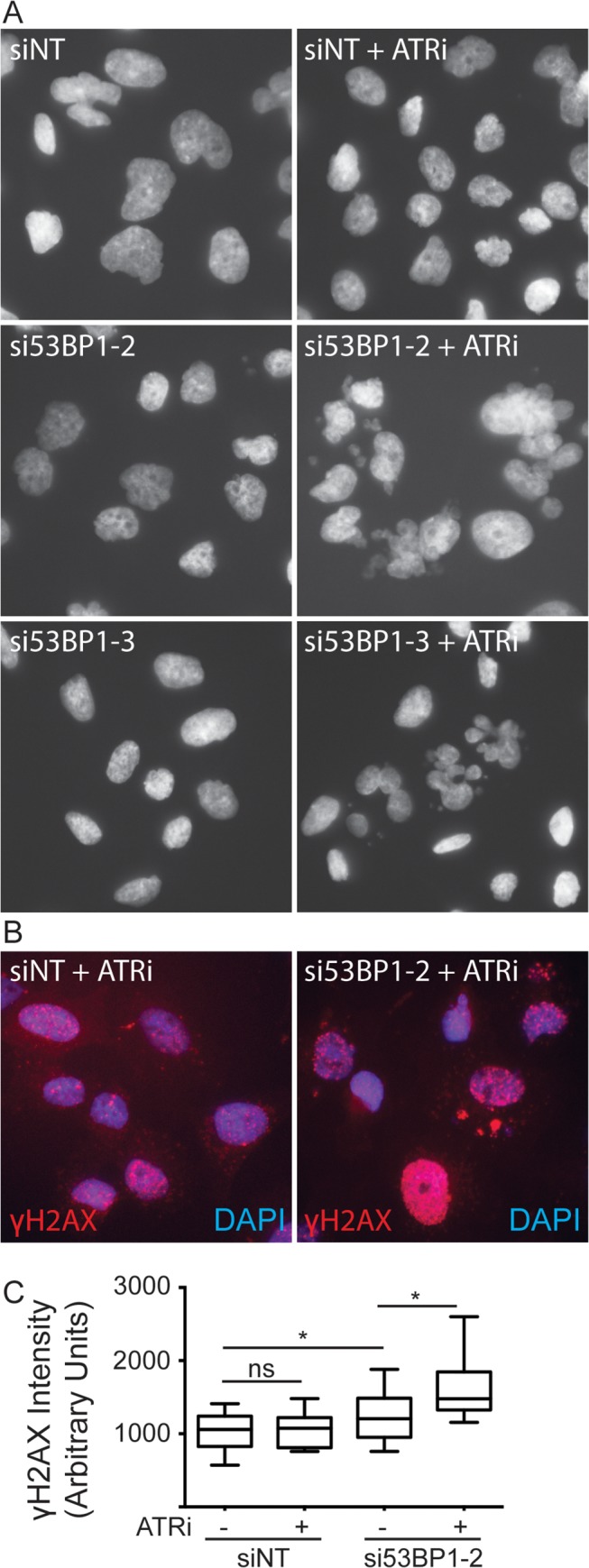
ATR is required to maintain genome integrity in 53BP1-deficient cells. H157 cells were transfected with non-targeting (siNT) or 53BP1 siRNA and then treated with 2.5μM ATRi for 24 hours. Cells were then fixed and stained with DAPI to visualize nuclei (A) and γH2AX to identify sites of DNA damage (B). (C) Quantification of the γH2AX intensity of cells shown in B. Box and whiskers plot shows the mean and the range of the samples, * p<0.01, ns (not significant).

The observed synthetic lethality between ATR inhibition as a single-agent and loss of 53BP1 was examined to determine which function of 53BP1 was required to promote cell viability. 53BP1 is required to protect under-replicated areas of the genome during mitosis [44, 45]. These areas are often present at common fragile sites and at areas of replication stress, such as those induced with ATR inhibition. To test whether 53BP1 was required to protect these sites we transfected H157 cells with non-targeting or 53BP1-specific siRNA, treated them with ATRi for 24 hours, and looked for any aberrant nuclear structures. Cells depleted of 53BP1 and treated with ATRi showed a dramatic increase in the number of failed mitoses as evidenced by nuclear fragmentation, micronuclei, and anaphase bridges (Fig 9A). None of these phenotypes were observed in control cells treated with the ATR inhibitor. 53BP1-depleted cells also showed an increase in γH2AX staining when treated with the ATR inhibitor, indicative of DNA damage (Fig 9B and 9C). Thus 53BP1 is likely required to protect under-replicated or damaged DNA induced by ATRi inhibition.

## Discussion

ATR inhibitors are in clinical trials for the treatment of many forms of cancer and show strong synergy with cisplatin and other DNA damaging chemotherapy agents. To identify genetic determinants of response we conducted a synthetic lethal siRNA screen with ATRi/cisplatin combination to identify genes, that when lost, sensitize cancer cells to this treatment. We screened a library of DNA damage and replication genes and identified several synthetic lethal interactions. ATR inhibition alone synergizes best with loss of ATR pathway genes, DNA replication genes, ERCC1, and ribonucleotide reductase. The ATR/cisplatin combination synergizes best with loss of translesion DNA polymerases and 53BP1. Furthermore, ATRi exhibits strong synergy with PARP inhibitors. Importantly, we observe no synthetic lethality with loss of HR or MMR suggesting that tumors with these defects are unlikely to respond better to combination treatments containing the ATR inhibitor.

### Loss of TLS improves response to ATR inhibition and cisplatin

One of the novel gene families identified in this screen is TLS polymerases. These specialized polymerases are required to incorporate nucleotides opposite damaged bases to allow for continued DNA replication. Loss of Pol ζ was the strongest scoring synthetic lethal interaction after *POLD2* and *ATR*. NSCLC and TNBC cells depleted of the catalytic subunit, REV3, showed synthetic lethality with ATRi alone and marked synergy with ATRi/cisplatin treatment in every cell line tested, further validating our observation. Pol ζ is viewed as a master regulator in TLS, as Pol ζ and REV1 are required to complete repair initiated by several other TLS polymerases [40]. In addition to its role in cisplatin resistance, REV3 is also required for maintaining viability after replication fork stalling and for preventing expression of common fragile sites [46, 47]. Loss of REV3 also causes persistent DNA damage and an increase in γH2AX [48]. Inhibiting any of these functions would create an increased need for ATR activity to maintain genomic stability and cell viability. The treatment of REV3-deficient cells with ATRi/cisplatin likely creates DNA inter- and intra-strand crosslinks that cannot be replicated past as well as expression of common fragile sites that could collapse in mitosis to yield double strand breaks and aneuploidy, further compromising cell viability. Importantly, REV3 is mutated in greater than 17% of lung cancers in the cancer genome atlas (TCGA) data set [49]. REV3 is also located within a fragile site deleted in several types of human leukemias and solid tumors [50], and 7% of B cell lymphomas in the TCGA data set have homozygous deletions in REV3 [49] making the synthetic lethal relationship with ATRi/cisplatin potentially useful clinically.

### Loss of 53BP1 improves response to ATRi/cisplatin

*TP53BP1* was the second highest-scoring novel gene exhibiting synthetic lethality with ATRi/cisplatin treatment, after ATR pathway and DNA replication genes. 53BP1 loss in breast cancer correlates with TNBC status, loss of *BRCA* in hormone receptor positive breast cancer, and is associated with decreased overall survival [43, 51]. 53BP1 has many known function in DNA repair and influences the pathway choice between HR and non-homologous end joining. Work with PARP inhibitors indicates that 53BP1 is required for the synthetic lethality observed with PARP inhibitors and loss of *BRCA1/2*, as loss of 53BP1 rescues the observed synthetic lethality [41–43]. *BRCA1*-deficient tumors frequently lose 53BP1 to maintain cell viability [43, 51], thus limiting the potential effectiveness of selective PARP inhibitor treatment. We observed synthetic lethality with ATRi/cisplatin in U2OS cells and H157 NSCLC cell lines, as well as two TNBC cell lines. All of these cell lines tested are HR-proficient, suggesting that loss of 53BP1 will be synthetic lethal with ATRi/cisplatin in a wider set of genomic contexts.

53BP1 is also required to protect under-replicated regions of the genome, such as common fragile sites, during mitosis for repair in the subsequent G1. Cells depleted of 53BP1 and treated with ATRi exhibit a large increase in the amount of mitotic failures including fragmented nuclei, micronuclei, and anaphase bridges—all of which are problems associated with improper chromosome segregation. Therefore, the observed synthetic lethality likely arises from the critical need for 53BP1 to protect chromosomes with DNA damage problems in mitosis.

### Synthetic lethality with PARP inhibition

PARP inhibitors are currently in clinical trials, primarily for the targeted treatment of BRCA-deficient breast and ovarian cancers. ATR was previously validated as a major determinant of PARP inhibitor sensitivity in breast cancer and ovarian cancer [35, 36]. We demonstrate marked synergy between PARP and ATR inhibition, comparable to the synergy observed between ATRi and cisplatin. The PARP inhibitor BMN673 is 100-fold more potent at trapping PARP-DNA complexes than Olaparib or Veliparib [52] and functions by potently trapping PARP-DNA complexes, which are likely repaired by HR during DNA replication [26, 34]. Our data indicates that ATR is also required for the repair of these trapped complexes. The synergy between these two drugs may re-sensitize BRCA-deficient tumors that do not respond to PARP inhibition alone, and allow the larger population of BRCA-proficient tumors to benefit from this combination therapy. However, this combination treatment could lose the specificity for cancer cells over normal cells.

In summary, we report an analysis of synthetic lethality of DNA repair pathways with ATRi/cisplatin combination treatment. The work presented here identifies novel synthetic lethal interactions that can be utilized for patient selection and improved predictive outcomes as ATR inhibitors progress through clinical trials.

## Supporting Information

S1 Dataset Results from the sensitivity screens. This table includes the siRNA sequences used to target each gene in the library and the robust z-scores for each siRNA for each replicate of the screen. Calculation of the robust z-scores is described in the Materials and Methods. The ATRi single-agent screen was previously published and included for comparison to the other drug treatments [18].
(XLS)

S1 Fig Related to Fig 7. Loss of REV3 is synthetic lethal with ATRi and cisplatin. (A-G) U2OS cells were transfected with non-targeting siRNA (siNT) or two siRNAs targeting REV3 (number 2 and 4 refer to specific sequences described in the materials and methods). Cells were then treated with ATRi, cisplatin, and ATRi and cisplatin. Cell viability was determined with alamar blue and reported as a percent of the untreated control cells. (A) Sensitivity of REV3 knockdown cells to cisplatin. (B and C) Sensitivity of REV3 knockdown cells to ATRi and ATRi with 0.1μM cisplatin. Bliss independence synergy between ATRi and cisplatin in control (D) and REV3 knockdown cells (E and F). (G) Isobologram analysis of synergy. (H) Cells were treated with 1μM ATRi, 0.1μM cisplatin, or both (A + C); cells were released into media without drugs after 24 hours and allowed to form colonies. Error bars in all panels are standard deviation (n = 3).
(TIF)

S2 Fig Related to Fig 7. Loss of REV3 is synthetic lethal with ATRi and cisplatin. (A-F) A549 NSCLC cells were transfected with non-targeting siRNA (siNT) or two siRNAs targeting REV3 (number 2 and 4 refer to specific sequences described in the materials and methods). Cells were then treated with ATRi, cisplatin, and ATRi and cisplatin. Cell viability was determined with alamar blue and reported as a percent of the untreated control cells. (A) Sensitivity of REV3 knockdown cells to cisplatin. (B and C) Sensitivity of REV3 knockdown cells to ATRi and ATRi with 0.1μM cisplatin. Bliss independence synergy between ATRi and cisplatin in control (D) and REV3 knockdown cells (E and F). Error bars in all panels are standard deviation (n = 3).
(TIF)

S3 Fig Related to Fig 7. Loss of REV3 is synthetic lethal with ATRi and cisplatin. (A-F) HCC1806 TNBC cells were transfected with non-targeting siRNA (siNT) or two siRNAs targeting REV3 (number 2 and 4 refer to specific sequences described in the materials and methods). Cells were then treated with ATRi, cisplatin, and ATRi and cisplatin. Cell viability was determined with alamar blue and reported as a percent of the untreated control cells. (A) Sensitivity of REV3 knockdown cells to cisplatin. (B and C) Sensitivity of REV3 knockdown cells to ATRi and ATRi with 0.1μM cisplatin. Bliss independence synergy between ATRi and cisplatin in control (D) and REV3 knockdown cells (E and F). Error bars in all panels are standard deviation (n = 3).
(TIF)

S4 Fig Related to Fig 7. Loss of REV3 is synthetic lethal with ATRi and cisplatin. (A-F) BT549 TNBC cells were transfected with non-targeting siRNA (siNT) or two siRNAs targeting REV3 (number 2 and 4 refer to specific sequences described in the materials and methods). Cells were then treated with ATRi, cisplatin, and ATRi and cisplatin. Cell viability was determined with alamar blue and reported as a percent of the untreated control cells. (A) Sensitivity of REV3 knockdown cells to cisplatin. (B and C) Sensitivity of REV3 knockdown cells to ATRi and ATRi with 0.5μM cisplatin. Bliss independence synergy between ATRi and cisplatin in control (D) and REV3 knockdown cells (E and F). Error bars in all panels are standard deviation (n = 3).
(TIF)

S5 Fig Related to Fig 8: Isobologram analysis of synergy in H157 using the dose response curve data in Fig 8. (TIF)

S6 Fig Related to Fig 8: Loss of 53BP1 is synthetic lethal with ATRi and cisplatin. (A-F) A549 NSCLC cells were transfected with non targeting siRNA (siNT) or two siRNAs targeting 53BP1 (number 2 and 3 refer to specific sequences described in the materials and methods). Cells were then treated with ATRi, cisplatin, and ATRi and cisplatin. Cell viability was determined with alamar blue and reported as a percent of the untreated control cells. (A) Sensitivity of 53BP1 knockdown cells to cisplatin. (B and C) Sensitivity of 53BP1 knockdown cells to ATRi and ATRi with 0.5μM cisplatin. Bliss independence synergy between ATRi and cisplatin in control (D) and 53BP1 knockdown cells (E and F). Error bars in all panels are standard deviation (n = 3).
(TIF)

S7 Fig Related to Fig 8: Loss of 53BP1 is synthetic lethal with ATRi and cisplatin. (A-F) HCC1806 TNBC cells were transfected with non targeting siRNA (siNT) or two siRNAs targeting 53BP1 (number 2 and 3 refer to specific sequences described in the materials and methods). Cells were then treated with ATRi, cisplatin, and ATRi and cisplatin. Cell viability was determined with alamar blue and reported as a percent of the untreated control cells. (A) Sensitivity of 53BP1 knockdown cells to cisplatin. (B and C) Sensitivity of 53BP1 knockdown cells to ATRi and ATRi with 0.5μM cisplatin. Bliss independence synergy between ATRi and cisplatin in control (D) and 53BP1 knockdown cells (E and F). Error bars in all panels are standard deviation (n = 3).
(TIF)

S8 Fig Related to Fig 8: Loss of 53BP1 is synthetic lethal with ATRi and cisplatin. (A-F) BT549 TNBC cells were transfected with non targeting siRNA (siNT) or two siRNAs targeting 53BP1 (number 2 and 3 refer to specific sequences described in the materials and methods). Cells were then treated with ATRi, cisplatin, and ATRi and cisplatin. Cell viability was determined with alamar blue and reported as a percent of the untreated control cells. (A) Sensitivity of 53BP1 knockdown cells to cisplatin. (B and C) Sensitivity of 53BP1 knockdown cells to ATRi and ATRi with 0.5μM cisplatin. Bliss independence synergy between ATRi and cisplatin in control (D) and 53BP1 knockdown cells (E and F). Error bars in all panels are standard deviation (n = 3).
(TIF)
