# Supplementary figures and images for: A Synthetic Lethal Screen Identifies DNA Repair Pathways that Sensitize Cancer Cells to Combined ATR Inhibition and Cisplatin Treatments
Source: PLoS One. 2015 May 12;10(5):e0125482. doi: 10.1371/journal.pone.0125482 (PMC4428765; doi:10.1371/journal.pone.0125482)

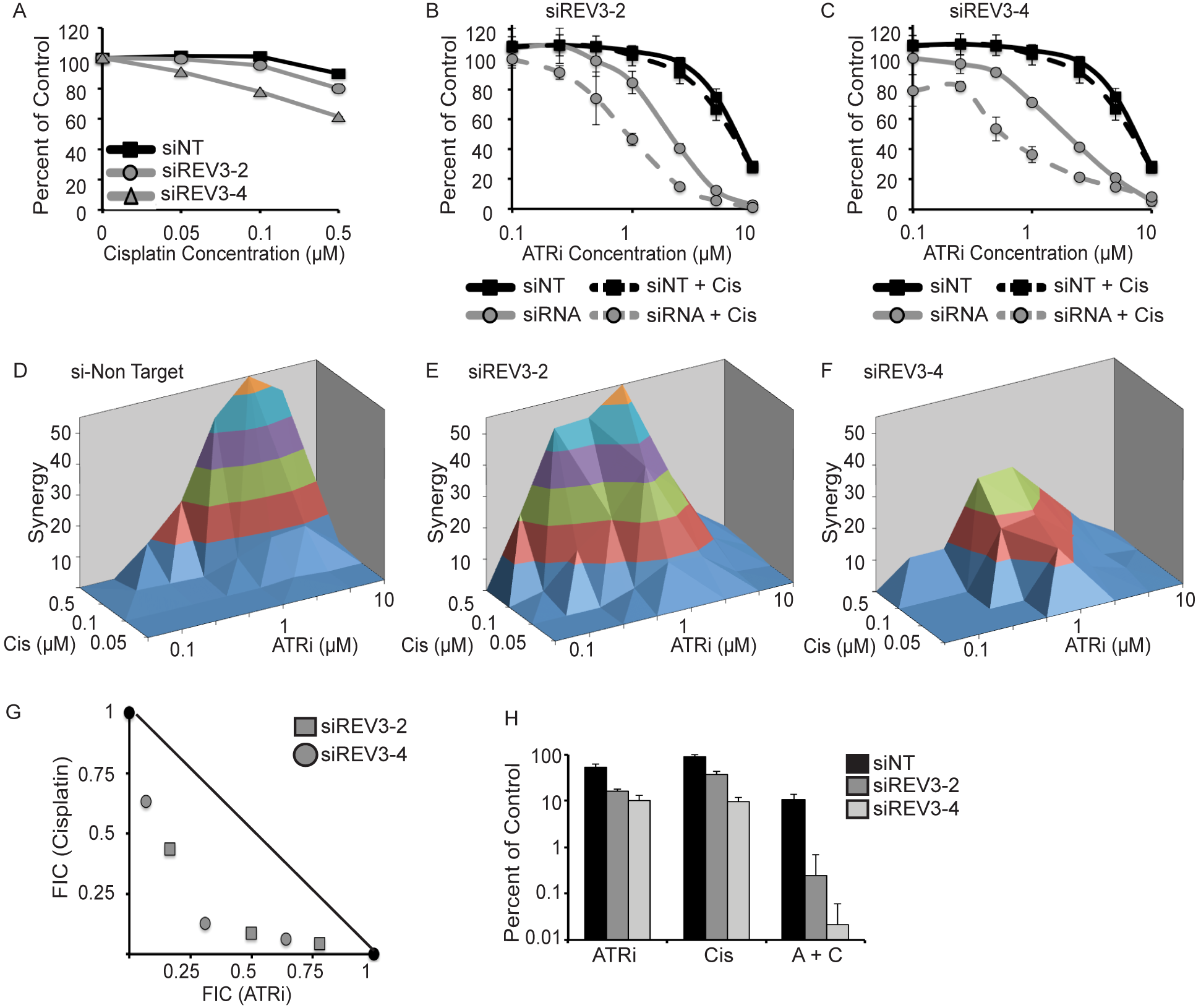

Supplement: S1 Fig — Loss of REV3 is synthetic lethal with ATRi and cisplatin. (A-G) U2OS cells were transfected with non-targeting siRNA (siNT) or two siRNAs targeting REV3 (number 2 and 4 refer to specific sequences described in the materials and methods). Cells were then treated with ATRi, cisplatin, and ATRi and cisplatin. Cell viability was determined with alamar blue and reported as a percent of the untreated control cells. (A) Sensitivity of REV3 knockdown cells to cisplatin. (B and C) Sensitivity of REV3 knockdown cells to ATRi and ATRi with 0.1μM cisplatin. Bliss independence synergy between ATRi and cisplatin in control (D) and REV3 knockdown cells (E and F). (G) Isobologram analysis of synergy. (H) Cells were treated with 1μM ATRi, 0.1μM cisplatin, or both (A + C); cells were released into media without drugs after 24 hours and allowed to form colonies. Error bars in all panels are standard deviation (n = 3). (TIF) [file pone.0125482.s002.tif]

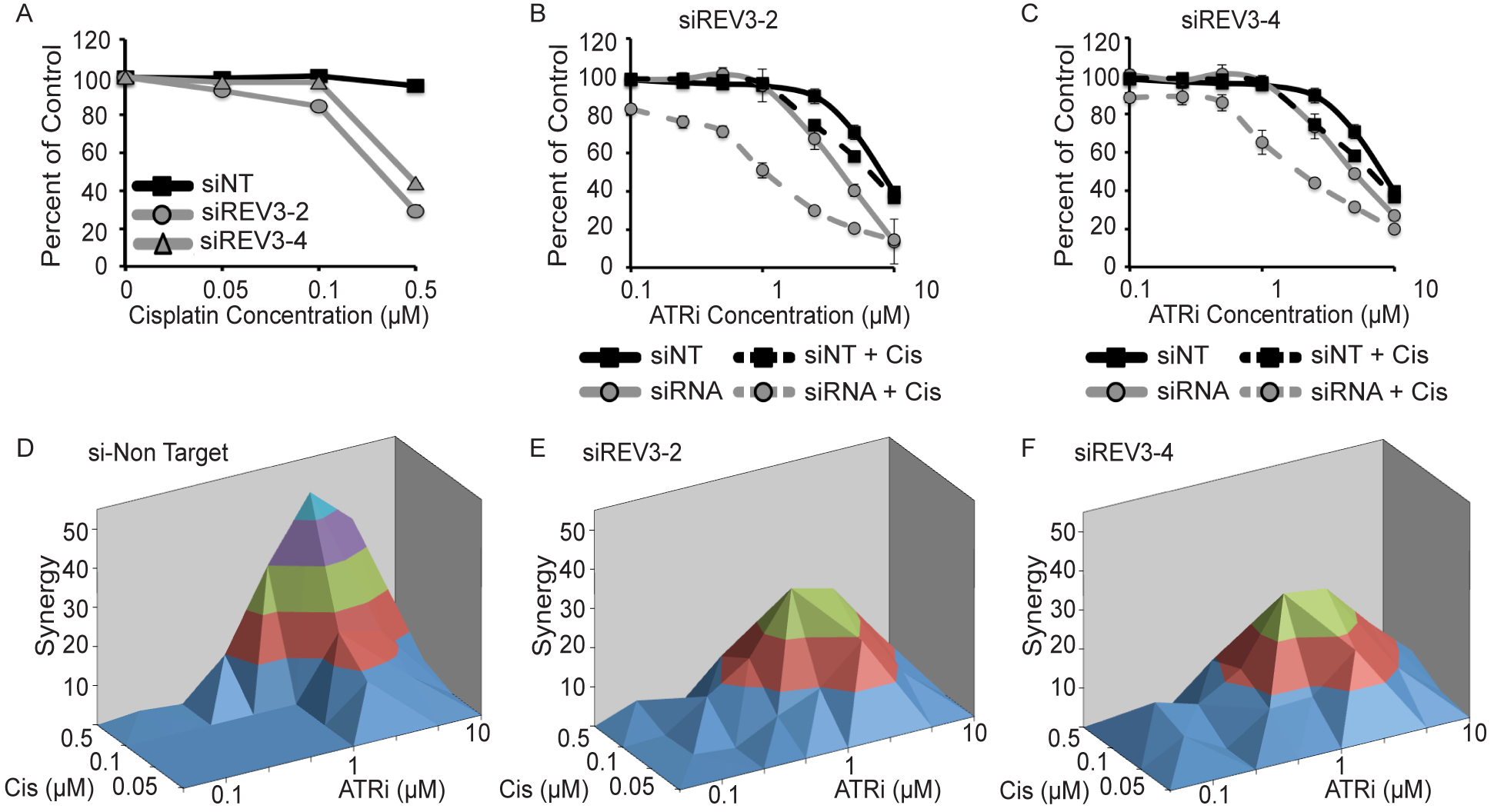

Supplement: S2 Fig — Loss of REV3 is synthetic lethal with ATRi and cisplatin. (A-F) A549 NSCLC cells were transfected with non-targeting siRNA (siNT) or two siRNAs targeting REV3 (number 2 and 4 refer to specific sequences described in the materials and methods). Cells were then treated with ATRi, cisplatin, and ATRi and cisplatin. Cell viability was determined with alamar blue and reported as a percent of the untreated control cells. (A) Sensitivity of REV3 knockdown cells to cisplatin. (B and C) Sensitivity of REV3 knockdown cells to ATRi and ATRi with 0.1μM cisplatin. Bliss independence synergy between ATRi and cisplatin in control (D) and REV3 knockdown cells (E and F). Error bars in all panels are standard deviation (n = 3). (TIF) [file pone.0125482.s003.tif]

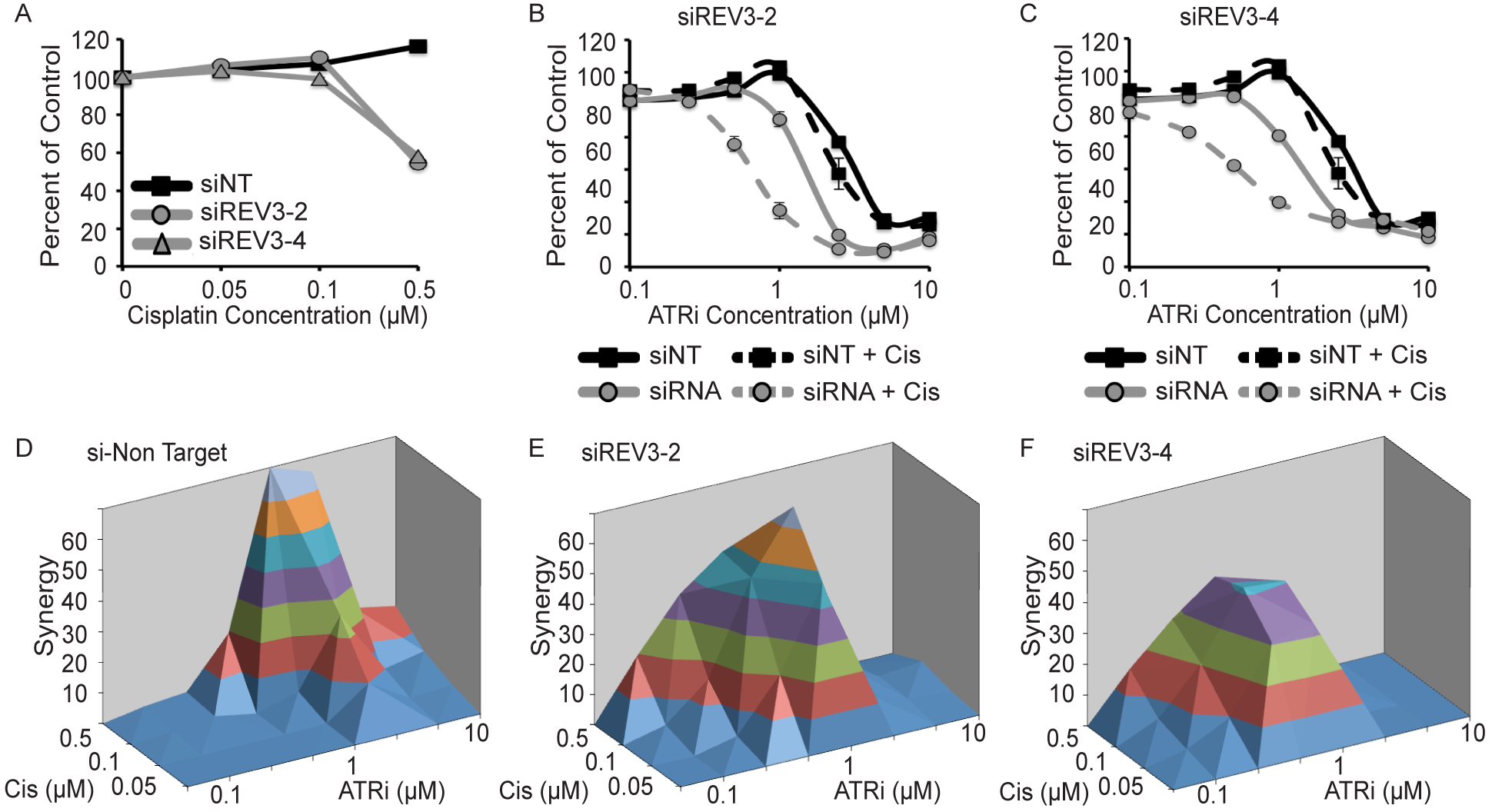

Supplement: S3 Fig — Loss of REV3 is synthetic lethal with ATRi and cisplatin. (A-F) HCC1806 TNBC cells were transfected with non-targeting siRNA (siNT) or two siRNAs targeting REV3 (number 2 and 4 refer to specific sequences described in the materials and methods). Cells were then treated with ATRi, cisplatin, and ATRi and cisplatin. Cell viability was determined with alamar blue and reported as a percent of the untreated control cells. (A) Sensitivity of REV3 knockdown cells to cisplatin. (B and C) Sensitivity of REV3 knockdown cells to ATRi and ATRi with 0.1μM cisplatin. Bliss independence synergy between ATRi and cisplatin in control (D) and REV3 knockdown cells (E and F). Error bars in all panels are standard deviation (n = 3). (TIF) [file pone.0125482.s004.tif]

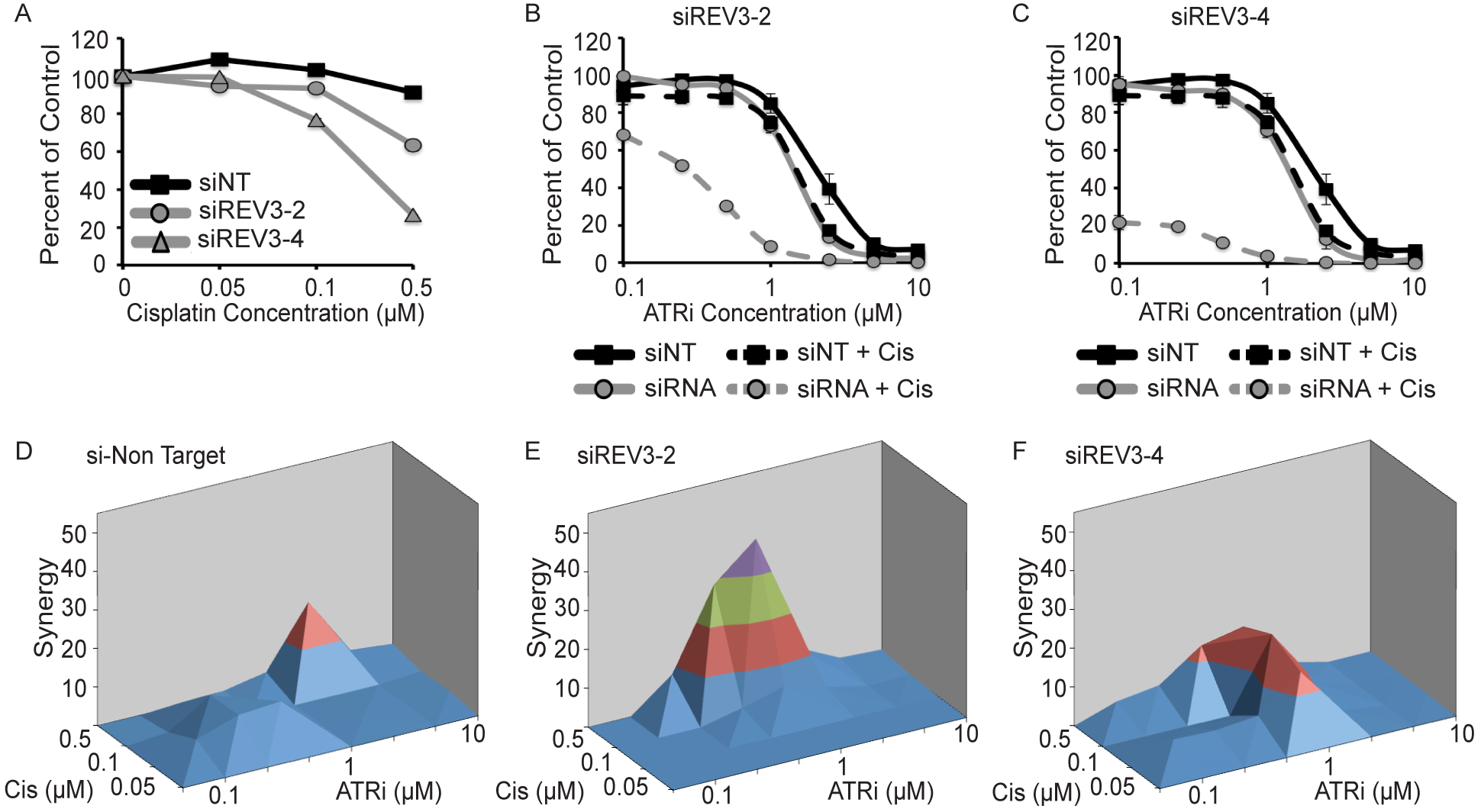

Supplement: S4 Fig — Loss of REV3 is synthetic lethal with ATRi and cisplatin. (A-F) BT549 TNBC cells were transfected with non-targeting siRNA (siNT) or two siRNAs targeting REV3 (number 2 and 4 refer to specific sequences described in the materials and methods). Cells were then treated with ATRi, cisplatin, and ATRi and cisplatin. Cell viability was determined with alamar blue and reported as a percent of the untreated control cells. (A) Sensitivity of REV3 knockdown cells to cisplatin. (B and C) Sensitivity of REV3 knockdown cells to ATRi and ATRi with 0.5μM cisplatin. Bliss independence synergy between ATRi and cisplatin in control (D) and REV3 knockdown cells (E and F). Error bars in all panels are standard deviation (n = 3). (TIF) [file pone.0125482.s005.tif]

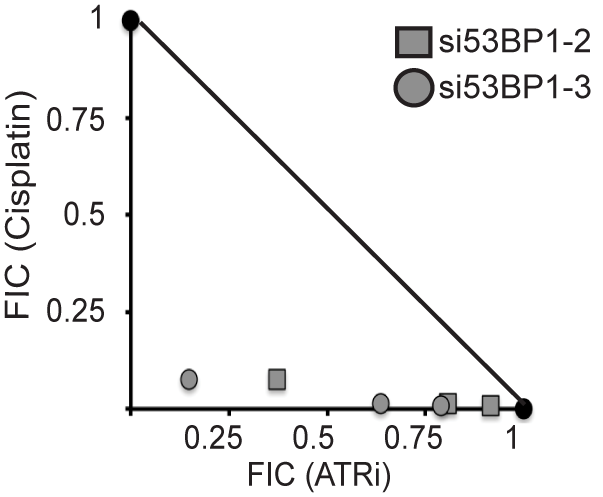

Supplement: S5 Fig — (TIF) [file pone.0125482.s006.tif]

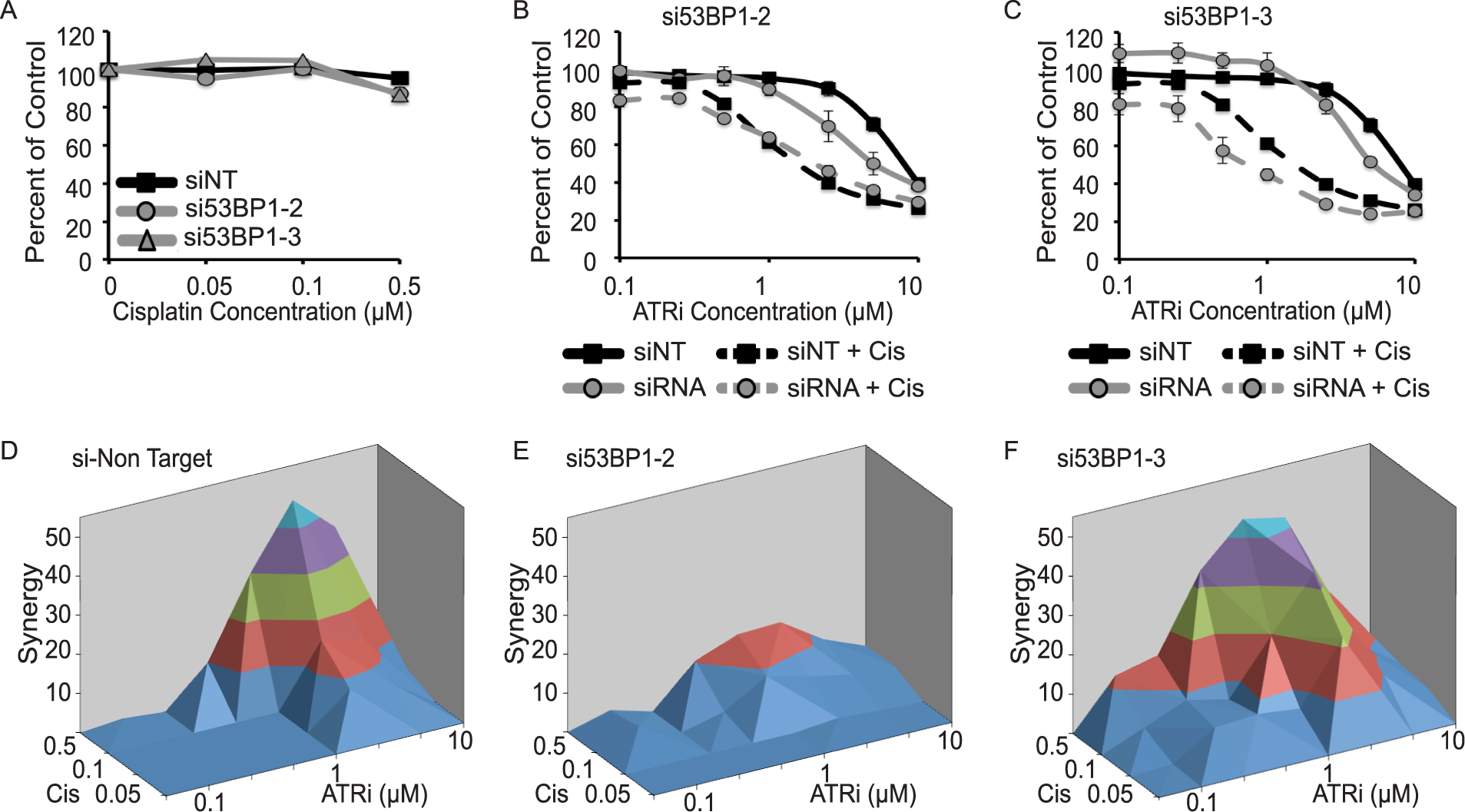

Supplement: S6 Fig — (A-F) A549 NSCLC cells were transfected with non targeting siRNA (siNT) or two siRNAs targeting 53BP1 (number 2 and 3 refer to specific sequences described in the materials and methods). Cells were then treated with ATRi, cisplatin, and ATRi and cisplatin. Cell viability was determined with alamar blue and reported as a percent of the untreated control cells. (A) Sensitivity of 53BP1 knockdown cells to cisplatin. (B and C) Sensitivity of 53BP1 knockdown cells to ATRi and ATRi with 0.5μM cisplatin. Bliss independence synergy between ATRi and cisplatin in control (D) and 53BP1 knockdown cells (E and F). Error bars in all panels are standard deviation (n = 3). (TIF) [file pone.0125482.s007.tif]

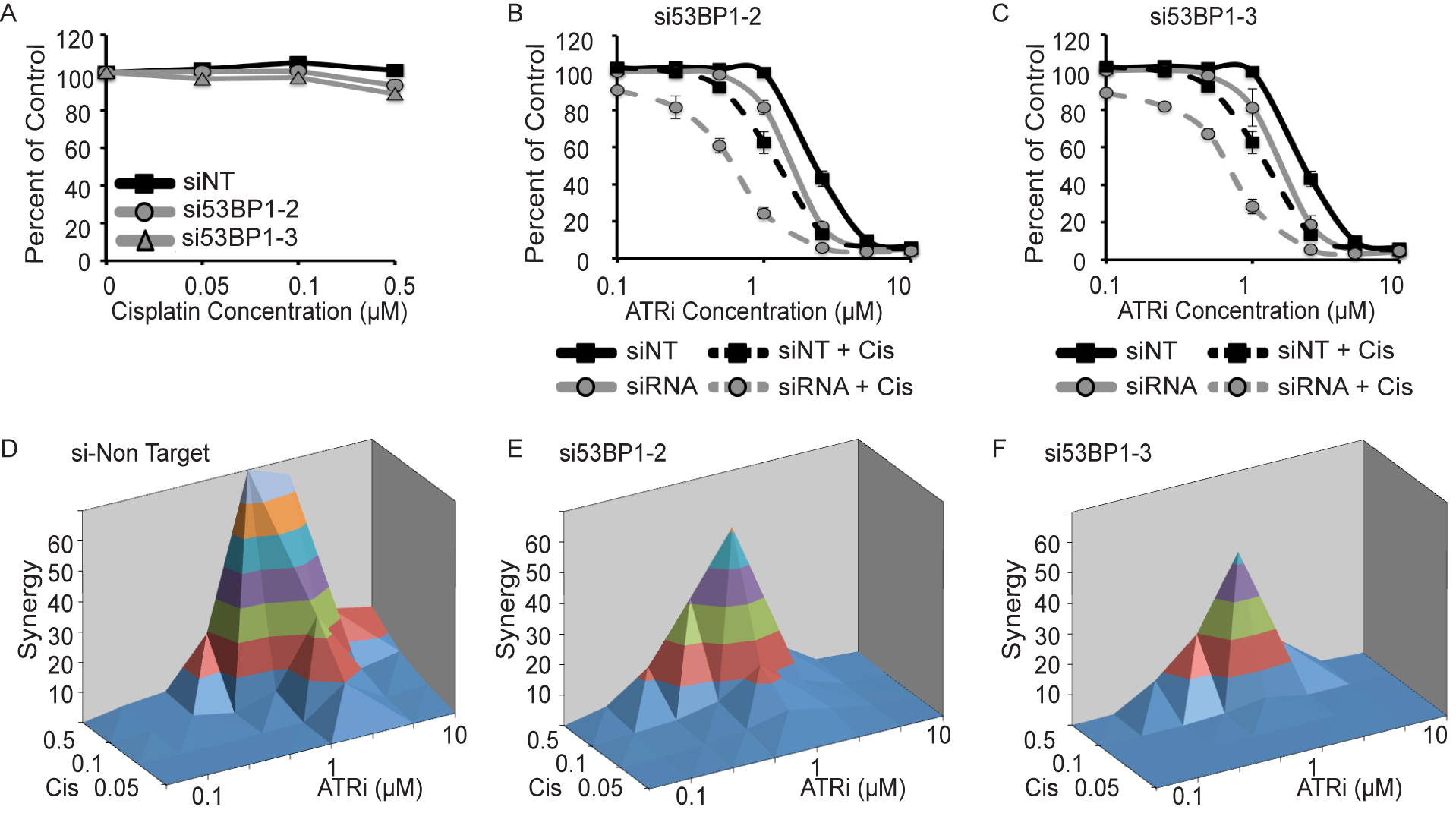

Supplement: S7 Fig — (A-F) HCC1806 TNBC cells were transfected with non targeting siRNA (siNT) or two siRNAs targeting 53BP1 (number 2 and 3 refer to specific sequences described in the materials and methods). Cells were then treated with ATRi, cisplatin, and ATRi and cisplatin. Cell viability was determined with alamar blue and reported as a percent of the untreated control cells. (A) Sensitivity of 53BP1 knockdown cells to cisplatin. (B and C) Sensitivity of 53BP1 knockdown cells to ATRi and ATRi with 0.5μM cisplatin. Bliss independence synergy between ATRi and cisplatin in control (D) and 53BP1 knockdown cells (E and F). Error bars in all panels are standard deviation (n = 3). (TIF) [file pone.0125482.s008.tif]

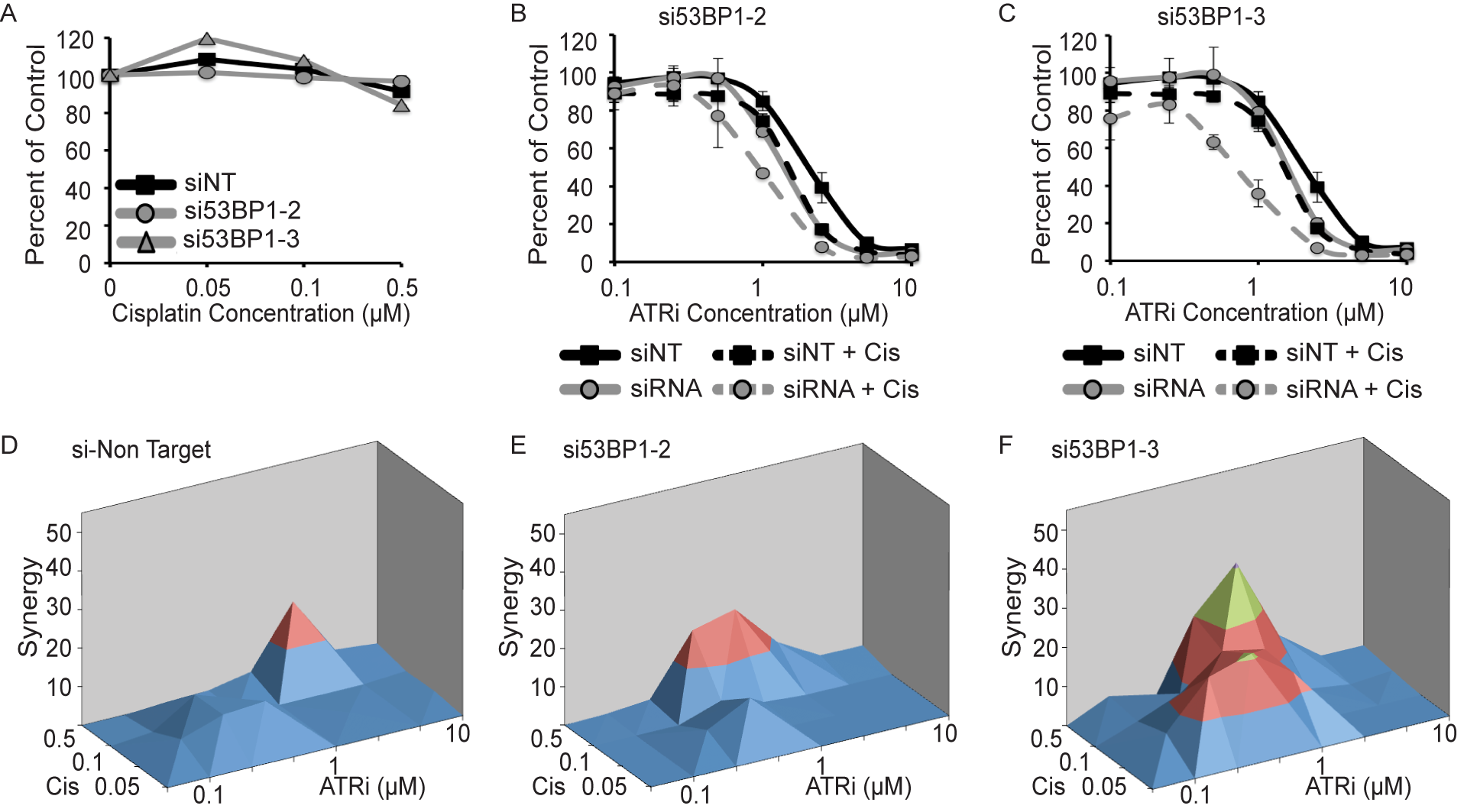

Supplement: S8 Fig — (A-F) BT549 TNBC cells were transfected with non targeting siRNA (siNT) or two siRNAs targeting 53BP1 (number 2 and 3 refer to specific sequences described in the materials and methods). Cells were then treated with ATRi, cisplatin, and ATRi and cisplatin. Cell viability was determined with alamar blue and reported as a percent of the untreated control cells. (A) Sensitivity of 53BP1 knockdown cells to cisplatin. (B and C) Sensitivity of 53BP1 knockdown cells to ATRi and ATRi with 0.5μM cisplatin. Bliss independence synergy between ATRi and cisplatin in control (D) and 53BP1 knockdown cells (E and F). Error bars in all panels are standard deviation (n = 3). (TIF) [file pone.0125482.s009.tif]
